# Neutrophil Activation and Early Features of NET Formation Are Associated With Dengue Virus Infection in Human

**DOI:** 10.3389/fimmu.2018.03007

**Published:** 2019-01-11

**Authors:** Anunya Opasawatchai, Panicha Amornsupawat, Natnicha Jiravejchakul, Wilawan Chan-in, Nicholas J. Spoerk, Khajohnpong Manopwisedjaroen, Pratap Singhasivanon, Tawatchai Yingtaweesak, Swangjit Suraamornkul, Juthathip Mongkolsapaya, Anavaj Sakuntabhai, Ponpan Matangkasombut, Fabien Loison

**Affiliations:** ^1^Department of Microbiology, Faculty of Science, Mahidol University, Bangkok, Thailand; ^2^Faculty of Dentistry, Mahidol University, Bangkok, Thailand; ^3^Systems Biology of Diseases Research Unit, Faculty of Science, Mahidol University, Bangkok, Thailand; ^4^Department of Clinical Pathology, Faculty of Medicine Vajira Hospital, Navamindradhiraj University, Bangkok, Thailand; ^5^Department of Bacteriology, College of Agricultural and Life Sciences, University of Wisconsin-Madison, Madison, WI, United States; ^6^Department of Tropical Hygiene, Faculty of Tropical Medicine, Mahidol University, Bangkok, Thailand; ^7^Thasongyang Hospital, Tak, Thailand; ^8^Endocrine Unit, Department of Medicine, Vajira Hospital, Bangkok, Thailand; ^9^Department of Medicine, Imperial College London, London, United Kingdom; ^10^Dengue Hemorrhagic Fever Research Unit, Office for Research and Development, Faculty of Medicine, Siriraj Hospital, Mahidol University, Bangkok, Thailand; ^11^Functional Genetics of Infectious Diseases Unit, Institut Pasteur, Paris, France; ^12^Centre National de la Recherche Scientifique (CNRS), URA3012, Paris, France

**Keywords:** innate immunity, neutrophil, viral infection, dengue, NETs

## Abstract

The involvement of the immune system in the protection and pathology of natural dengue virus (DENV) has been extensively studied. However, despite studies that have referred to activation of neutrophils in DENV infections, the exact roles of neutrophils remain elusive. Here, we explored the phenotypic and functional responses of neutrophils in a cohort of adult dengue patients. Results indicated that during an acute DENV infection, neutrophils up-regulate CD66b expression, and produce a more robust respiratory response as compared with that in convalescent or healthy individuals; this confirmed *in vivo* neutrophil activation during DENV infection. Spontaneous decondensation of nuclei, an early event of neutrophil extracellular trap (NET) formation, was also markedly increased in cells isolated from DENV-infected patients during the acute phase of the infection. *In vitro* incubation of NETs with DENV-2 virus significantly decreased DENV infectivity. Interestingly, increased levels of NET components were found in the serum of patients with more severe disease form—dengue hemorrhagic fever (DHF), but not uncomplicated dengue fever, during the acute phase of the infection. Levels of pro-inflammatory cytokines IL-8 and TNFα were also increased in DHF patients as compared with those in healthy and DF subjects. This suggested that NETs may play dual roles during DENV infection. The increased ability for NET formation during acute DENV infection appeared to be independent of PAD4-mediated histone H3 hyper-citrullination. Our study suggests that neutrophils are involved in immunological responses to DENV infection.

## Introduction

The dengue virus (DENV) is a growing public health concern worldwide. There are four DENV serotypes, which cause a multitude of clinical outcomes that range from self-limiting febrile illness, dengue fever (DF), and dengue hemorrhagic fever (DHF) to life-threatening dengue shock syndrome (DSS) (1). The mechanisms underlying the pathogenesis of the disease and immune responses to DENV infection are not completely understood; however, these are likely the result of complex interplays between viral factors such as viral load, serotypes, and viral proteins (2, 3) and host factors, such as pre-existing immune responses and changes in endothelial cell composition (4–6).

The roles of neutrophils during viral infections have been re-evaluated following the discovery of neutrophil extracellular traps (NETs). NET formation (NETosis) consists of a series processes characterized by nuclear decondensation and delobulation, rupture of the plasma membrane, and release of DNA fibers with antimicrobial peptides (7). While NETosis is a potent anti-microbial mechanism, excessive formation of NETs, or the inability to clear NETs from the circulation, contributes to pathogenesis of autoimmune diseases (8). The roles of NETs in viral infections vary greatly from virus to virus. Beneficial roles of NETs in trapping (HIV) (9) and inhibiting viral dissemination (murine pox virus) (10) have been reported. NETs and neutrophil-derived products have been found in lungs of influenza virus-infected mice (11). However, inhibition of NET formation did not affect infection outcomes (12). On the contrary, excessive NET formation resulted in airway obstruction during respiratory syncytial virus (RSV) infection (13) and exacerbated allergic airway inflammation during rhinovirus (RV) infection (14). Moreover, components of NETs were shown to serve as autoantigens in patients infected with the hemorrhagic hantavirus (15). Interestingly, the mechanisms underlying NET formation and the factors that dictate the role of NETs during viral infections remain largely unknown.

Converging data suggest that neutrophils are not simple bystanders during DENV infection. While it is known that DENV-infected patients exhibit sustained neutropenia, the clinical relevance of this phenomenon is still poorly understood (16). Increased levels of pro-inflammatory mediators such as IL-8 and TNFα can activate neutrophils (4). In addition, anaphylatoxins (17) were found in acute and severe dengue patients. Importantly, expression of genes associated with neutrophil function (DEF4A, CEACAM8, BPI, and ELA2) was found to be increased in whole blood transcriptomes of severe DENV infections (18). A recent study showed that neutrophil elastase levels were higher in DENV-infected patients as compared with those in healthy controls (19). DHF patients also had significantly higher level of elastase activity as compared with that of DF patients, which suggested that neutrophil activation is associated with severe form of the disease (19). Intriguingly, another recent study showed that DENV could induce formation of NETs *in vitro* (20). However, due to their short half-lives, the study of neutrophil functions, NETs, and their association with disease severity in naturally-infected dengue patients is challenging, and to our knowledge, have never been reported.

Here, neutrophils obtained from DENV-infected patients were collected, processed, and analyzed in a timely manner. We performed a longitudinal study that examined neutrophil phenotypes and functional responses across different severity levels of DENV infection. Our results were the first to show neutrophil activation and their susceptibility to NET formation; our study highlighted possible roles of neutrophils during human DENV infections. Our findings provide new understanding of host immune responses during DENV infections by targeting potential roles of neutrophils.

## Materials and Methods

### Ethics Statement

All participants included in the present study were adults; written informed consents were provided by all subjects prior to study onset. Blood samples were collected from adult dengue patients and healthy individuals at the Vajira Hospital and the Tropical Medicine Hospital in Bangkok, as well as the Thasongyang Hospital in Tak province, Thailand. Ethical approval was obtained from ethics committees at the Vajira Hospital and the Tropical Medicine Hospital, Mahidol University (2013-046-03).

### Study Cohort, Blood Sample Collection, and Neutrophil Isolation

Index cases were included based on positive nested-RT-PCR for dengue viral RNA (serotype 1, 2, 3, or 4). Severity of DENV infection was classified into DF or DHF according to WHO criteria (1). Household members who did not show signs of DENV infection, and for which DENV could not be detected by nested-RT-PCR in the serum, served as healthy controls. The number of patients and control subjects used at least once for each assay are presented in Table S1. Blood samples from index cases were collected daily from admission until day of defervescence, and were further classified according to the phase of infection using clinical symptoms. Febrile samples (Feb DENV) refer to specimen collected on days of high fever until the fever subsided (Defervescence, Def DENV). Convalescent samples (Con DENV) were collected 2 weeks following the first admission, when patients were fully recovered. All functional *ex vivo* experiments were performed on the day of blood collection. For western blot, neutrophil pellets were dried and stored at −80°C. In addition, serum was prepared and stored at −80°C. To isolate neutrophils, fresh heparinized blood samples were centrifuged (22°C, 800 g, 10 min) to separate cells from plasma. Cell suspensions were diluted in RPMI-1640 medium supplemented with 2% fetal bovine serum (FBS) (Gibco, MA, USA) before isolation on an Isoprep layer (Robbins Scientific Corporation, CA, USA). The pellet containing red blood cells (RBCs) and granulocytes was subjected to RBC lysis via the addition of a hypotonic NaCl solution (0.2%), cells were incubated for 30 s before adding an equal volume of 1.6% NaCl. Recovered cell pellets were washed and resuspended in RPMI 1640 completed with 0.5% FBS. Giemsa staining and FACS staining were performed and showed that the pellets contained routinely more than 95% neutrophils.

### Flow Cytometry

White blood cells were separated from heparinized whole blood samples using RBC lysis buffer (Biolegend, CA, USA). After washing with PBS, recovered white blood cells were incubated with fluorophore-conjugated antibodies against CD11b (PEcy7, #557743, BD Pharmigen) and CD66b (FITC, #555724, BD Pharmigen), or the corresponding isotype controls (BD Bioscience). Samples were acquired on a BD FACS Canto II. Granulocytes were gated using FSC/SSC, as well as double expression of CD11b and CD66b. Data were analyzed using the Flowjo v.8.7 software (Treestar, USA). Delta mean fluorescence intensity (MFI) was determined by subtracting the background fluorescence of the isotype control from specific MFIs of each pair of antibodies.

### Detection of Reactive Oxygen Species *ex vivo*

The level of neutrophil intracellular reactive oxygen species (ROS) was measured *ex vivo* by flow cytometry using dihydrorhodamine (DHR) 123 (Invitrogen, MA, USA). DHR 123 is converted into green fluorescent rhodamine 123 by hydrogen peroxide. Following RBC lysis, fresh heparinized blood samples were incubated in the presence of DHR 123 for 15 min at 37°C, with or without PMA. ROS level was determined based on green fluorescence intensity of neutrophils gated on FSC/SSC using flow cytometry.

### Immunofluorescence

The protocol for NET visualization by immunofluorescence was as previously described (15, 21). Briefly, 2 × 10^5^ cells in RPMI-1640-0.5% FBS were seeded onto a round glass coverslip in a 24-well plate. Cells were allowed to adhere for 10 min before addition of PMA at various concentrations for 2 h (37°C, 5% CO_2_). Cells were then fixed with 4% paraformaldehyde, and were permeabilized with PBS containing Triton-X 100 (0.5%). Nuclear morphology and NETs were stained using Sytox Green (1 μM, ThermoFisher, MA, USA), Hoechst 3342, or Hoechst and an anti-MPO antibody (Santa Cruz, sc-52707, 1:500). Cells were mounted onto slides, and were observed using a Nikon Eclipse 80i microscope. The percentage of NET formation was quantified by counting cells with decondensed/rounded nuclei and extracellular Sytox green positive fibers in at least three randomly pictured fields.

### MPO-DNA ELISA

MPO-DNA ELISA was adapted from Caudrillier et al. (22). Briefly, flat bottom 96-well plates (Thermofisher Scientific, MA, USA) were coated with an anti-MPO polyclonal antibody (1:500, #2329755, Millipore, CA, USA) at 4°C overnight. Plates were washed with 0.05% Tween in PBS three times before addition of 20 μL patient serum pre-mixed with anti-DNA peroxidase antibody (1:80, Cell Death ELISA Plus kit, Roche, Germany). Cells were allowed to incubate at room temperature in the dark for 3 h under agitation. Plates were then washed five times prior to addition of the substrate Reserve™ TMB (KPL, PA, USA). The reaction was stopped by adding an equal amount of 1 N HCl; O.D. (450 nm) was measured using a microplate reader (EZ400, Biochrom, UK).

### Detection of Plasma Cytokines

EDTA plasma (25 μL) from healthy controls, DF, and DHF patients with acute infection were analyzed for a panel of 38 cytokines including IL-8 and TNFα by Luminex bead-based multiplex assay using the Milliplex® MAP human cytokine/chemokine magnetic bead panel kit (Millipore, USA). The FlexMAP 3D (Luminex®) platform was used according to manufacturer's instructions.

### Neutrophil Elastase ELISA

Plasma level of Neutrophil Elastase was measured with a Human PMN Elastase ELISA Kit (Abcam) following manufacturer's instruction. Briefly, standard human NE and 1:100 plasma samples were incubated on 96-well plate pre-coated with polyconal antibody against human NE in duplicates. The NE was then probed by the addition of HRP-conjugated anti-Human alpha1-P1 polyclonal antibody. After the addition of TMB for 20 min, followed by stop solution, the O.D. was read at 450 nm using a microplate reader (EZ400, Biochrom, UK). The average reading between duplicate wells subtracted by blank was used to calculate the plasma NE concentration (ng/ml).

### Cell Lysis and Histone Extraction

Neutrophils were frozen immediately after isolation, and were kept at −80°C until use. Frozen cell pellets were lysed in low salt buffer (20 mM Hepes, 1 mM Sodium EDTA, 1 mM Sodium EGTA, 500 mM KCl, 50 mM PMSF, and 75 mM MgCl_2_) in a protease inhibitor cocktail (cOmplete ULTRA, Roche, USA). After centrifugation (16,000 g, 4°C, 15 min), pellets containing the nuclear and membrane fractions were treated overnight with 0.5 N HCl at 4°C to extract histones. Histones present in the HCl soluble fraction were then precipitated with TCA acid (33.33%), and were washed with cold acetone. The acid-insoluble fractions were also collected for detection of PAD4. Protein concentration was determined by the Bradford protein assay (Biorad, CA, USA).

### Detection of Citrullinated Histone H3 and PAD4 by Western Blot

Histone extracts were separated on a 14% acid-urea gel, as previously described (23). Proteins were then transferred onto a PVDF membrane in 0.7% acetic acid buffer. Alternatively, acid-insoluble nuclear fractions were separated by 10% SDS-PAGE before transferring onto a PVDF membrane using a semi-dry blotting system (Biorad, CA, USA). Membranes were blocked with 5% skimmed milk in TBS-0.2% Tween for 1 h, and were incubated with rabbit anti-citrullinated histone3 antibodies (1:2,000, #ab5103, Abcam, Cambridge, UK) for histone extracts or mouse anti-PAD4 antibodies (1:2,000, #ab57167, Abcam, Cambridge, UK) for acid-insoluble nuclear fractions. Membranes were stripped and probed with rabbit anti-total histone3 (Abcam, #1791) for loading control. Chemiluminescence was captured using a GBox (Syngene, Cambridge, UK), and band intensity was quantified by densitometry using the ImageJ software (NIH).

### Cell Culture and DENV Propagation

C6/36 cells were maintained at 25°C in L-15 medium supplemented with tryptose phosphate broth and 10% FBS. At 80–90% confluency, DENV-2 diluted in 2% FBS L-15 (MOI:0.01) was added to the C6/36 monolayer at room temperature under gentle agitation. The cell monolayer was washed to remove unbound viruses prior to addition of fresh 2% FBS L-15. Cell supernatant was harvested 3 and 5 days post-infection, and the viral titer was determined by FFU assay.

### Coincubation of Neutrophils With DENV *in vitro*

Neutrophils isolated from healthy volunteers (2 × 10^5^) were placed onto a glass coverslip in a 24-well plate, and were incubated with 500 μL RPMI-2% autologous serum for 10 min. The supernatant was removed prior to addition of the virus (DENV2, MOI 1:1). Alternatively, neutrophils were plated for 10 min before PMA (100 nM) was added for 3 h to induce NET formation. The supernatant was then carefully removed, and the virus was added. The virus was incubated for 10, 30, or 180 min with neutrophils or NETs. At the end of the incubation period, supernatants were collected, and viral titers were determined by FFU. Coverslips were recovered, fixed (4% paraformaldehyde), and stained with Hoechst 33342 (Thermo-Fisher Scientific).

### Foci Forming Unit (FFU) Assay

BHK-21 cells were maintained at 37°C in DMEM with 10% FBS. Ten-fold serial dilutions of C6/36 cell supernatants were incubated with 3 × 10^5^ BHK cells in 96-well plates at 37°C for 90 min. This was followed by addition of 1.5% carboxymethyl cellulose (CMC), and cells were incubated at 37°C for 3 days. After fixation with 3.7% paraformaldehyde, cells were permeabilized with 0.1% TritonX and washed with PBS. DENV-specific anti-E antibody (4G2) was added for 3 h at 37°C before washing with PBS. Cells were stained with anti-mouse Ig secondary antibody tagged with HRP for 45 min at 37°C, and washed three more times with PBS. Diammonium phosphate (DAP) was added at room temperature for color visualization; FFU were counted under a light microscope.

### Statistical Analysis

Statistical analyses were performed using the Prism version 5.0 software. The Mann-Whitney U-test was a used to compare the median between two different groups of donors (e.g., healthy control vs. DENV infected patients). The Wilcoxon-signed rank test was used to compare the median obtained from the same patient at two different time points (e.g., febrile vs. convalescent phase). Differences between more than two groups of samples were determined by Kruskal–Wallis tests, followed by Dunn's *post-hoc* tests. Correlation between the two sets of data obtained from the same donor (e.g., level of CD66b expression vs. percent spontaneous delobulation) was evaluated by the Spearman-rank correlation test.

## Results

### Neutrophils Were Stimulated During Acute DENV Infection

**Figure 1:**
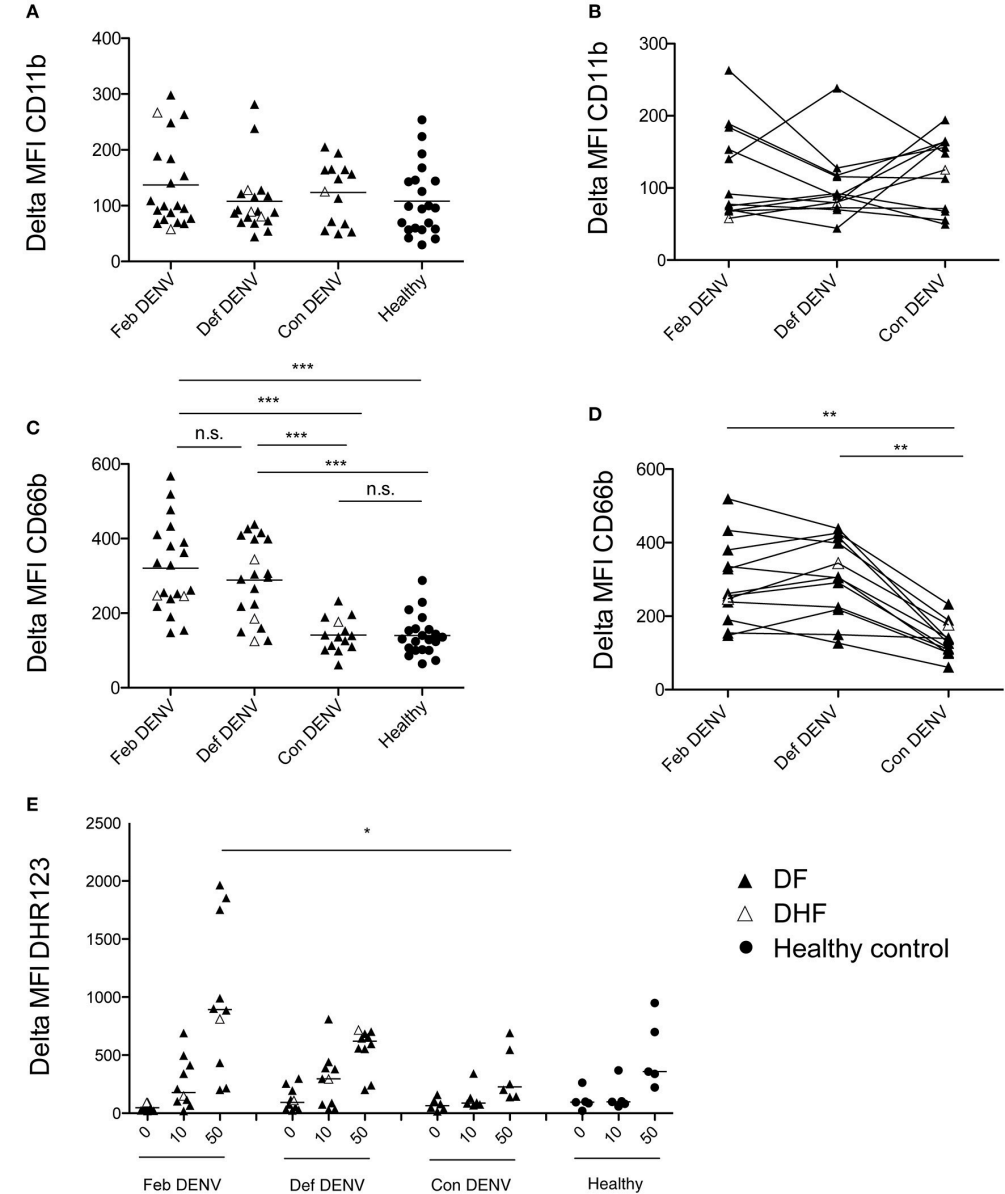
Neutrophil activation during acute DENV infection. Delta MFI of CD11b **(A)** and CD66b **(C)** in healthy donors (*n* = 21), during febrile illness (Feb DENV) (*n* = 21), deferevescence (Def DENV) (*n* = 20), and convalescence (Con DENV) (*n* = 14). Bars represent the mean of each population. ***p < 0.001, as assessed by the Kruskal-Wallis test, followed by Dunn's *post-hoc* test. Neutrophil CD11b **(B)** and CD66b **(D)** surface expression at the different phase of the disease in individual patients. **p < 0.01, assessed by the Wilcoxon-signed rank test. **(E)** Granulocytes were gated by size and granularity prior to ROS production analysis by flow cytometry. Dot plots show level of ROS production by the mean fluorescent intensity of DHR-123 in Feb DENV (*n* = 10), Def DENV (*n* = 10), Con DENV (*n* = 6), and healthy controls (*n* = 5). *p < 0.05, assessed by the Kruskal–Wallis test, followed by Dunn's *post-hoc* test. In all panels, DF patients are represented by a dark arrowhead, DHF patients by a white arrowhead, and healthy control by a dark circle.

To identify and assess the phenotype of neutrophils in whole blood, we measured expression levels of the activation markers CD11b and CD66b by flow cytometry (24, 25). No significant change was observed in CD11b expression (Figures 1A,B). However, CD66b expression was markedly increased in febrile patients (Feb DENV) as compared with that of healthy controls (*p* < 0.001) (Figure 1C); this difference was maintained until defervescence (Def DENV, Figure 1D). At convalescence, CD66b level was significantly decreased (*p* < 0.001), and was comparable to that of healthy donors (Figure 1C). The DENV infection-associated CD66b upregulation was found in both mild (DF) and severe (DHF) patients (Figures 1C,D). We did not observe any correlation between CD66b expression and viral titer or virus serotype (data not shown). In line with CD66b upregulation, PMA induced a stronger respiratory burst in neutrophils from febrile patients as compared with that in convalescent and healthy individuals (Figure 1E), respiratory bursts are characteristic of primed neutrophils (26). Altogether, these results suggested that neutrophils are activated during acute DENV infections.

### Neutrophil Activation During DENV Infection Resulted in Increased Nuclei Delobulation

**Figure 2:**
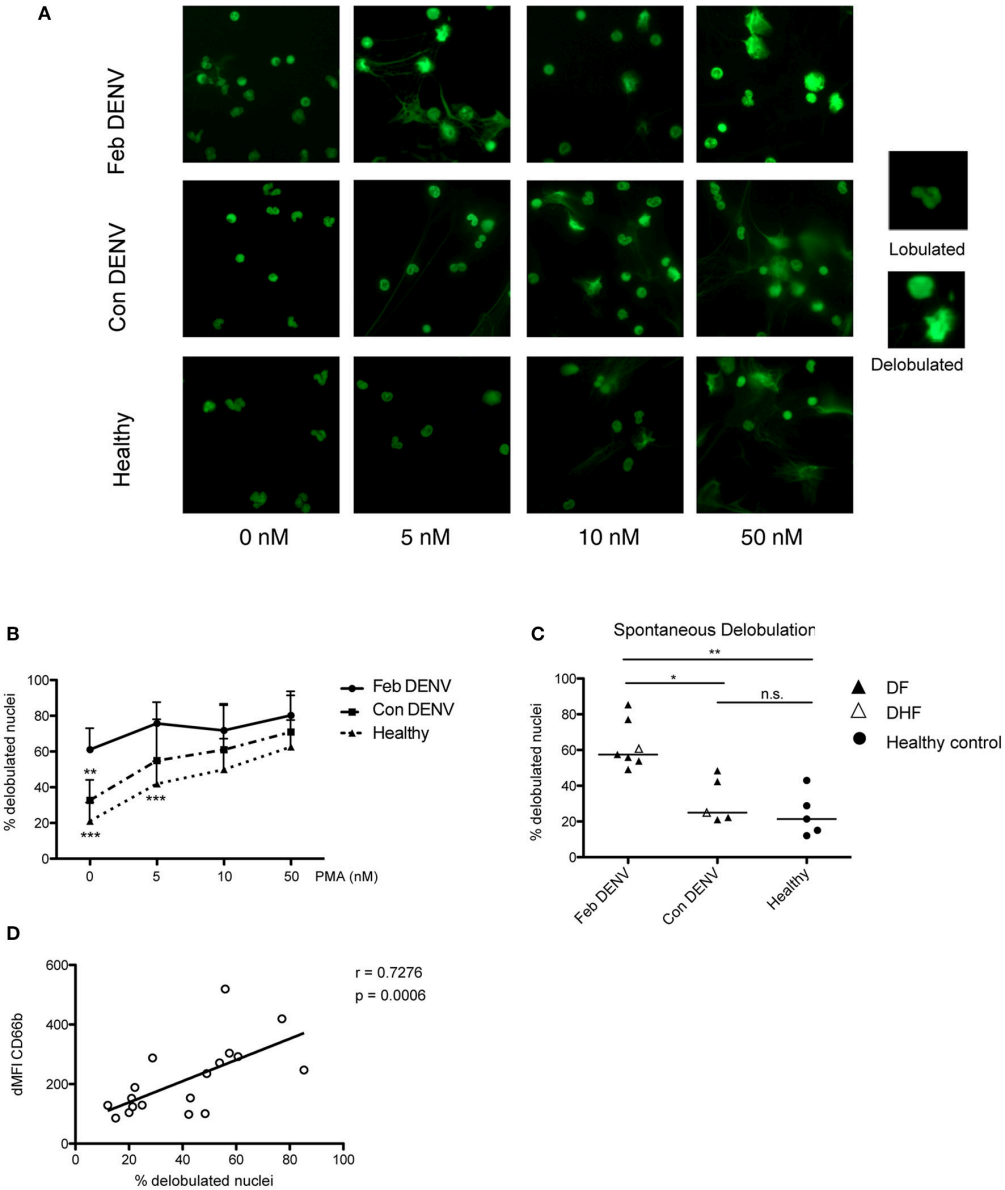
Neutrophils from acute DENV infected patients are susceptible to NET formation. Isolated neutrophils from healthy donors and DENV infected patients during febrile illness (Feb DENV) and convalescent phase of the disease (Con DENV) were cultured for 2 h without stimulation or with 5, 10, or 50 PMA. The cells were fixed, permeabilized, and stained with the DNA dye Sytox green, and were visualized with a fluorescent microscope. **(A)** Representative images of nuclear morphologies of isolated neutrophils from Feb DENV, Con DENV, and healthy donors in response to varying concentration of PMA stimulation. Representative high magnifications of lobulated and delobulated nuclei are shown on the right. **(B)** Average percentage of cells with delobulated nuclei from Feb DENV (*n* = 7), Con DENV (*n* = 5), and Healthy donors (*n* = 5) in response to varying PMA concentration. Error bars represent mean ± SEM, **p < 0.01, ***p < 0.001, 2way ANOVA with Bonferroni's multiple comparisons test. **(C)** Percentage of cells undergoing nuclei decondensation without PMA stimulation from Feb DENV (*n* = 7), Con DENV (*n* = 5), and healthy donors (*n* = 5). Bars represent mean of each group. *p < 0.05, **p < 0.01, analyzed by Kruskal–Wallis test followed by Dunn's *post-hoc* test. **(D)** Correlation between cells with decondensed nuclei with CD66b expression in matched donors, as determined by Spearman-ranked correlation (*r* = 0.620, *p* = 0.0010, *n* = 17).

ROS are required for NET formation in response to various stimuli (27, 28). As high levels of cell-free DNA are associated with severe DENV infection (29), and elevated levels of NET components, myeloperoxidase, and neutrophil elastase, have been found during the acute phase of the infection (18, 19), we hypothesized that neutrophils form NETs during DENV infections. We found that nuclei of unstimulated healthy neutrophils remained lobulated after 2 h, as did the cells for all conditions before incubation (Figure S1). In contrast, nuclei of unstimulated neutrophils from acute, but not convalescent, patients were delobulated after 2 h (*p* < 0.01) (Figures 2A,C). Moreover, the percentage of delobulated cells was greater in acute DENV neutrophils as compared with that of healthy and convalescent cells for each PMA concentration tested (Figure 2B). The presence of PMN with delobulated nuclei was not due to contaminating cells as shown by FACS and Giemsa staining prior incubation (Figure S1). In healthy human neutrophils, the increased number of cells with delobulated nuclei was associated with the release of NETs in unstimulated cells and in response to LPS and PMA (Figure S2). The loss of lobes has been shown to be an early mark of NET formation (27, 28, 30). This observation suggested a possible increased propensity for acute DENV neutrophils to form NETs. Interestingly, cell expression of CD66b and percentage of nuclei lobule loss were positively correlated in matched donors (*p* < 0.01) which suggested that CD66b may be an early marker of NETs during DENV infection (Figure 2D). No association was observed between the expression of CD11b or CD16 and spontaneous delobulation (data not shown). To assess if PMN delobulation and activation were observable *in vivo*, the SSC values of neutrophils for healthy controls and DENV patients obtained at different time were analyzed. The SSC mean fluorescence intensity for febrile DENV patient neutrophils (442.7 ± 72.62) was lower compared to healthy controls (481.7 ± 89.74), but the difference was not statistically different (Figure S3).

### NETs Impaired DENV Infectivity *in vitro*

**Figure 3:**
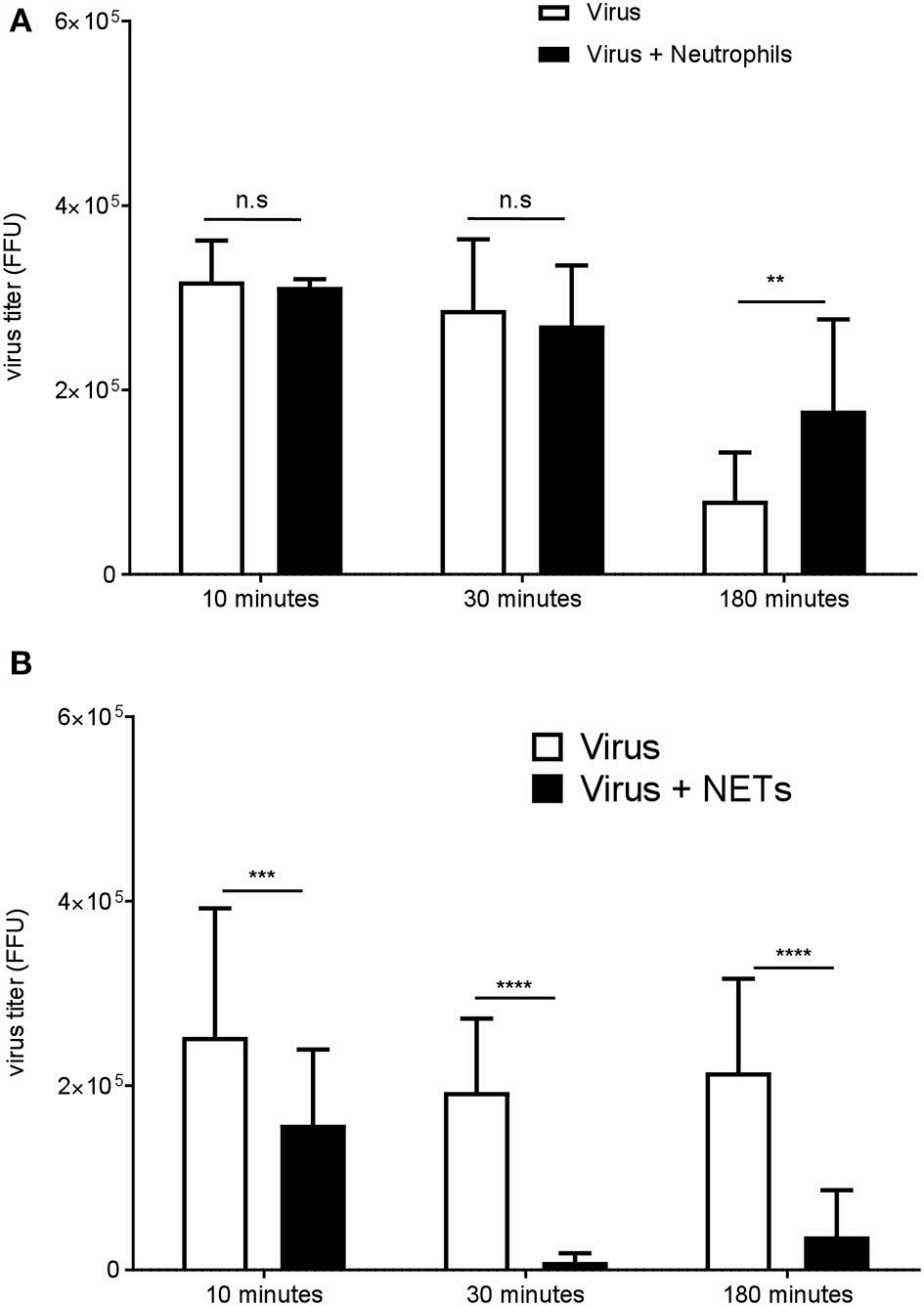
NETs impair DENV infectivity *in vitro*. Neutrophils from healthy donors were cultured in the presence **(A)** or absence of PMA (100 nM) **(B)** for 3 h to induce NETs formation. After removal of the supernatant, the virus was added for 10, 30, or 180 min to neutrophils, NETs, or empty well controls. The virus titer recovered after incubation with neutrophils **(B)** or with NETs **(A)** was determined by the FFU assay. Data are shown means ± SD (*n* = 3). **p < 0.01, ***p < 0.0001,****p < 0.0001, adjusted *p* values, 2way ANOVA with Bonferroni's multiple comparisons test.

NETs have been shown to trap and inhibit viruses *in vitro*, and can prevent viral dissemination *in vivo* (10, 31). As DENV neutrophils were prone to spontaneous formation of NETs *in vitro*, we investigated whether PMA-induced NETs from healthy neutrophils could inactivate DENV (Figure 3A). After 30 min of incubation, DENV titer was dramatically decreased in the presence of NETs as compared with that when incubated with the virus alone or in the presence of unstimulated healthy neutrophils (Figure 3B). This suggested that NETs could trap or inactivate the virus *in vitro*.

### Elevated IL-8 and TNFα Were Associated With Severe DENV Infection

**Figure 4:**
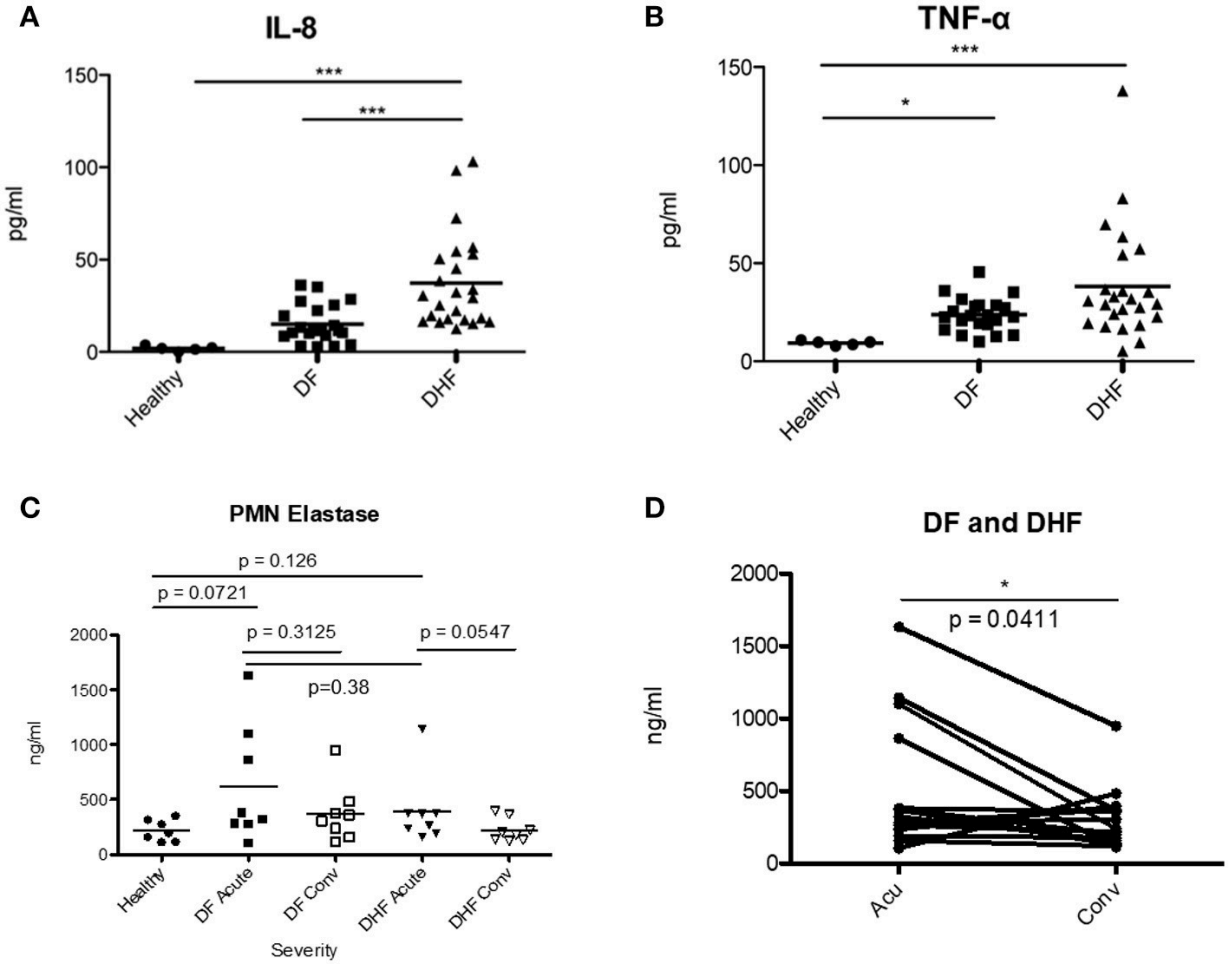
Elevated levels of IL-8, TNFα and Neutrophil Elastase during acute DENV infection. Plasma samples from healthy donors (healthy), mild (DF) and severe (DHF) DENV infected patients during febrile illness (Feb DENV) and convalescent phase (Con DENV) were used. **(A)** Plasma level of IL-8 and **(B)** TNFα during febrile DENV infection. *p < 0.05 and ***p < 0.001, Kruskal–Wallis test followed by Dunn's *post-hoc* test. **(C)** Level of NE (ng/ml) in the serum of healthy, DF and DHF patients during the febrile and convalescence phase. Mann–Whitney *U*-test. **(D)** Level of NE in the plasma of acute DENV patients (DF and DHF) vs. convalescent patients. NE concentration from the same patients are connected with lines. Wilcoxon-signed rank test, *p < 0.05.

To investigate whether neutrophil activation was correlated with a proinflammatory environment, we measured plasma levels of IL-8 and TNFα, cytokines known to activate neutrophils, during the febrile phase of DENV infection. We found significantly elevated IL-8 in DHF patients as compared with that of healthy controls (*p* < 0.001) and DF patients (*p* < 0.001) (Figures 4A,B). TNFα was significantly increased in DF and DHF patients as compared to healthy controls (*p* < 0.05, *p* < 0.001); however, no significant difference was found between DF and DHF patients. We also measured the level of Neutrophil Elastase, a maker of neutrophil activation, degranulation and NETosis, in the serum of patients (Figures 4C,D). We observed a slight increase of NE in the serum of DHF patients during the febrile phase as compared to convalescence, while the difference at the two time points was less obvious for DF patients (Figure 4C). The level of NE was significantly increased in Acute Dengue (DF and DHF) as compared to convalescence (*p* < 0.0411) (Figure 4D).

### Elevated NET-Derived Products Were Associated With Severe DENV Infection

**Figure 5:**
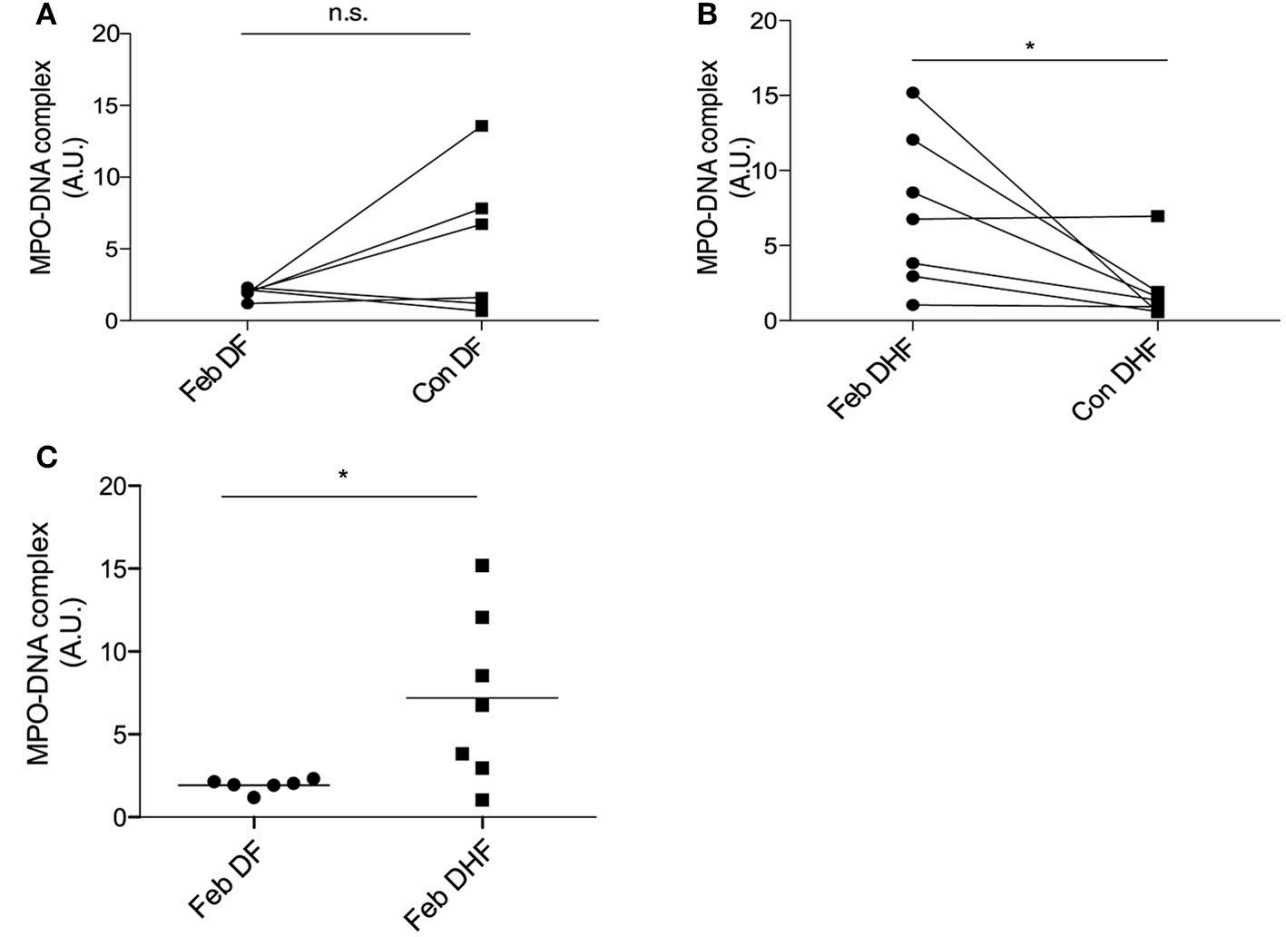
NETs are associated with severe DENV infection. Serum samples from healthy donors as well as mild (DF) and severe (DHF) DENV infected patients during febrile illness (Feb DENV) and convalescent phase (Con DENV) were used for the MPO-DNA ELISA. **(A,B)** O.D. ratios from the same DF and DHF patients are connected with lines. Analysis was performed with the Wilcoxon-signed rank test, *p < 0.05. **(C)** Each symbol represents O.D. at 450 nm normalized to inter-experimental control. Mann–Whitney *U*-test, *p < 0.05.

Nuclei delobulation (Figure 2) and change in SSC (Figure S3) were indirectly suggesting DENV neutrophils increased susceptible to NETosis. To determine whether NETs were produced during Dengue virus infection, and the association of NETs with clinical outcomes of DENV infection, we measured the level of MPO-DNA complexes in the serum of DENV-infected patients by ELISA (22). *Ex vivo* experiments indicated that febrile DENV infections increased susceptibility to spontaneous delobulation regardless of disease severity (Figure 2). However, serum levels of MPO-DNA complexes were elevated during febrile DENV infection in DHF patients, but not in DF patients, when compared with those in convalescent individuals (Figures 5A–C). Therefore, excessive NETs may be an aggravating factor that enhances disease severity during an acute DENV infection.

### Histone H3 Citrullination Was Suppressed During Acute DENV Infection

Protein arginine deiminase 4 (PAD4)-dependent histone citrullination is one of the pathway responsible for NET formation in microbial infections, systemic inflammations, and autoimmune diseases (32). We measured levels of Histone H3 citrullination (Figure S4) and PAD4 (Figure S5) in nuclear extracts of frozen neutrophils from DENV patients by western blot. Surprisingly, histone H3 citrullination was markedly decreased during the acute phase of DENV infection as compared with that during the convalescent stage and in healthy controls (Figure S4). PAD4 level was also slightly decreased during the acute phase of the infection compared with that during convalescence, albeit non-significant (Figure S5).

## Discussion

**Figure 6:**
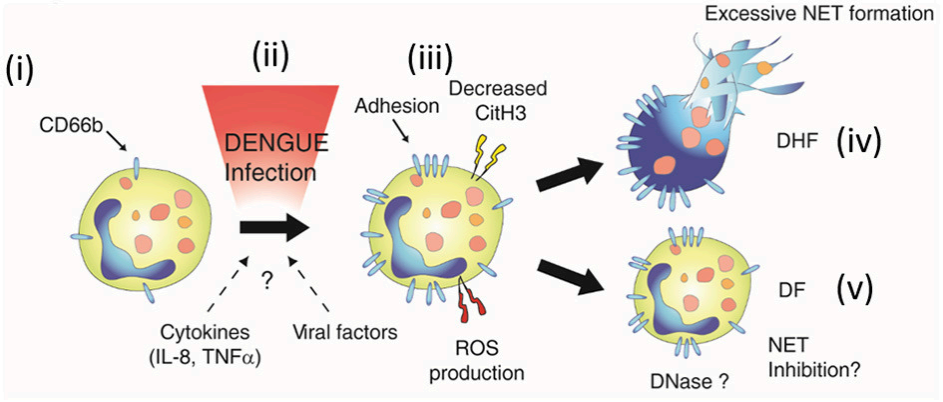
Neutrophil activation during DENV infection. During dengue infection, resting neutrophils **(i)** are activated **(ii)**. This activation is characterized by the upregulation of CD66b, increased production of ROS **(iii)**, and might be due to the proinflammatory environment (IL-8, TNFa), or unidentified viral or host factors **(ii)**. Neutrophil activation was also associated *ex vivo* with increased spontaneous delobulation for both DF and DHF patients (Figure 2). However, markers of NETs were found elevated only in the serum of the severe form of dengue (DHF, **iv**). This suggested that either NETosis is inhibited by a unknown mechanism or NETs degraded in the serum of DF patients (NET Inhibition and DNASe, respectively) **(v)**, and that these regulatory mechanisms are absent in DHF patients, leading to excessive NET formation **(iv)**.

Evidences of excessive inflammation during DENV infection suggested that activation of neutrophils and formation of NETs may be involved in disease pathogenesis (33). Due to access to neutrophil samples from DENV-infected patients, we assessed both the phenotype and function of neutrophils in naturally-infected DENV patients. We showed that neutrophils were primed *in vivo* (Figures 6i–iii), and were prone spontaneous delobulation. We also highlighted the dual roles of NETs in inhibiting DENV, as well as its possible contribution to disease severity.

Neutrophils overexpressed CD66b during acute DENV infection. CD66b is a marker of granulocyte activation involved in adhesion to endothelial cells (34), degranulation (35), and increased reactive oxygen species (ROS) production (34). Similar CD66b upregulation has been reported in patients during bacterial sepsis, a condition involving systemic inflammation (36). We did not observe CD11b overexpression associated with DENV infection. Interestingly, an increase in the CD66b:CD11b ratio has been reported for neutrophils in the presence of *S. aureus*, and this was associated with their reduced ability to phagocytosed (37). Our findings also confirm, at the protein level, the CD66b overexpression reported at the transcriptional level during DENV infection (18). In line with the elevated CD66b expression, we found robust ROS production by granulocytes in response to *ex vivo* PMA stimulation during acute dengue infection. This could be the result of priming by the pro-inflammatory environment found during acute dengue virus infection, as TNFα and IL-8, cytokines known to prime neutrophils for ROS production, were also found elevated in dengue patients as previously reported (38) and in our own study cohort (Figures 4A,B). ROS are essential both for neutrophil antimicrobial activity and for ROS-dependent NET formation (27, 39).

Emerging evidence has supported the presence and role of NETs in viral infections. Direct activation of neutrophils to form NETs by viral particles has been demonstrated in HIVs (31), RSVs (40), and hantaviruses (15). We provide evidences, in the present study, that during DENV infection, neutrophils are more susceptible to spontaneous delobulation *ex vivo*. Delobulation is an early feature of NET formation, but is not limited to the release of NETs and is found in other form of cell death. However, we confirmed that the limited spontaneous NETosis of healthy neutrophils, detected with an anti-PMO antibody, was also associated with the increased percentage of spontaneous delobulation (Figure S2). How DENV infection could result in such susceptibility remains to be elucidated. The ability of DENV particles to directly induce formation of NETs varies from one study to another. While Yost et al. showed that DENV could induce production of NETs in healthy neutrophils (20), Moreno-Altamirano et al. demonstrated that DENV-2 inhibited NET formation in response to PMA stimulation by altering the glycolysis pathway (41). In the present study, we could not characterize the mechanisms responsible for the elevated delobulation or the increase MPO-DNA complexes detected *in vivo* in natural DENV infection. However, we proposed that elevated levels of pro-inflammatory cytokines IL-8 and TNFα, which were reported to be inducers of NETs (7, 27, 42, 43) during febrile DENV infection, could contribute to such susceptibility through the priming or the activation of neutrophils [Figures 4A,B and (4). Neutrophil-platelet interactions may also promote NET formation during DENV infections (44)]; studies have shown that neutrophil-platelet interactions promoted production of NETs in the bacterial sepsis model (45) and murine pox virus infections (10). Furthermore, DENV has been shown to activate platelets (46, 47). Of note, we could not amplify viral genome from neutrophils isolated from dengue patients (data not shown).

NETs have been described as powerful antimicrobial weapons as well as threats to the host (48). In this study, we found higher susceptibility for NET formation in acute DENV infections regardless of disease severity. In addition, NETs could potentially inhibit DENV *in vitro*. Possible antiviral immunity elicited by NETs include immobilization of viral particles, inhibition of virus by antimicrobial proteins (31), and possibly through NET-induced type I interferon production by plasmacytoid dendritic cells (49). The precise mechanisms that underlie NET-mediated DENV inhibition need to be further investigated.

We detected elevated levels of Neutrophil Elastase in the plasma of Acute infected patients (Figures 4C,D). While not significant, the differences seemed more marked for DHF patients as compared to DF patients. NE elastase is a marker of neutrophil degranulation, and also found on NETs. Interestingly, higher levels of NET-derived components, such as the MPO-DNA complex, were also found in the serum of DHF patients as compared with that in DF patients (Figures 5A–C). Previously, two independent studies suggested that cell-free DNA (29) and plasma histone H2A (47), two other known components of NETs, were markedly elevated in severe form of DENV infection. These findings were in agreement with our observation, and showed that DENV infection may induce NET formation, and that elevated NET components are associated with severe disease.

The mechanisms underlying neutrophil activation and NET formation in dengue pathogenesis are currently unknown. Several studies have shown that canonical NETosis starts with the loss of neutrophil nucleus lobules (27, 28, 30). This is however not a proper marker of NETosis as other forms of cell death, such as leukotoxic hypercitrullination, can display similar features (50). However, the detection of NET products by ELISA in the serum of DENV patients, together with the low level of Histone citrullination, support an increased propensity of DENV neutrophils to release NETs. As we did not observe any difference in spontaneous formation of NETs between DF and DHF patients *ex vivo* (Figure 2), we propose that NET release may be inhibited, or that NETs were degraded in DF, but not DHF patients *in vivo* (Figures 6iv,v). It was proposed that metalloproteinases and histones in NETs may participate in vascular leakage in dengue infection by disrupting the vascular endothelial cell layer (51, 52). This suggest that the NET formation and degradation may affect the final outcomes of DENV infections. This phenomenon was also observed in SLE patients who exhibited excessive neutrophil-derived immune complex formation due to impairments in NET degradation (53).

Recent studies have suggested that PAD4 and histone citrullination-mediated NET formation is stimuli-dependent (50). Histone citrullination was found to be decreased and increased by PMA and ionomycin, respectively, however, both molecules stimulated NET formation (54). Little is known regarding NET formation in viral infections. However, PAD4-dependent NETs were not required for host protection during influenza infection *in vivo* (12). Similar to PMA-induced NET formation, we suggest that DENV-induced susceptibility to NET formation may be independent of PAD4 activity and histone H3 citrullination.

Targeting NETs using DNase have shown promising results in improving NET-mediated inflammation in the RV infection model (14) and in intestinal ischemic perfusion injury (55). Understanding the precise pathway responsible for NET formation in DENV infection, its regulation, and its impact on disease outcomes may shed light on potential therapeutic interventions for DENV infections in the future.

## Author Contributions

AO, PM, JM, AS, and FL designed the experiments. AO, PA, NS, NJ, and WC did the experiments. AO, PA, NS, and FL analyzed data. PM, PS, TY, SS, KM, and AS helped with sample collection and AO and FL wrote the manuscript.

### Conflict of Interest Statement

The authors declare that the research was conducted in the absence of any commercial or financial relationships that could be construed as a potential conflict of interest.

## Abbreviations

Feb DENV: Febrile phase of Dengue virus infection
Def DENV: Defervescent phase of Dengue virus infection
Con DENV: Convalescent phase of Dengue virus infection
Acu DENV: Acute phase of Dengue virus infection
tH3: Total histone H3
CitH3: Citrullinated histone H3.
