# Supplementary material for: Neutrophil Activation and Early Features of NET Formation Are Associated With Dengue Virus Infection in Human
Source: Front Immunol. 2019 Jan 11;9:3007. doi: 10.3389/fimmu.2018.03007 (PMC6336714; doi:10.3389/fimmu.2018.03007)
Supplement: Supplementary file 1 [file Presentation_1.pptx]

## Slide 1
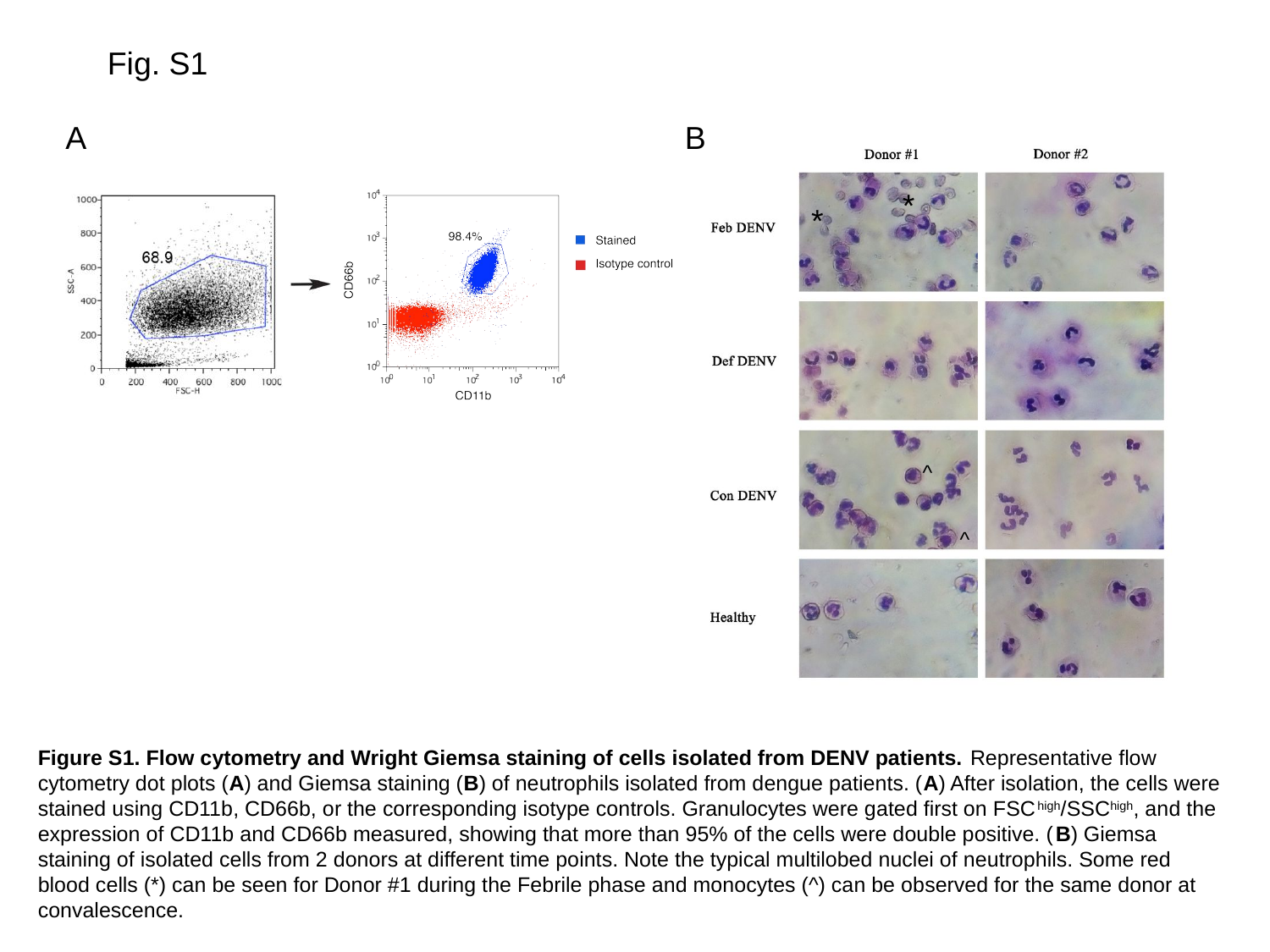

Fig. S1
A
B
*
*
*
^
^
^
^
Figure S1. Flow cytometry and Wright Giemsa staining of cells isolated from DENV patients. Representative flow cytometry dot plots (A) and Giemsa staining (B) of neutrophils isolated from dengue patients. (A) After isolation, the cells were stained using CD11b, CD66b, or the corresponding isotype controls. Granulocytes were gated first on FSChigh/SSChigh, and the expression of CD11b and CD66b measured, showing that more than 95% of the cells were double positive. (B) Giemsa staining of isolated cells from 2 donors at different time points. Note the typical multilobed nuclei of neutrophils. Some red blood cells (*) can be seen for Donor #1 during the Febrile phase and monocytes (^) can be observed for the same donor at convalescence.

## Slide 2
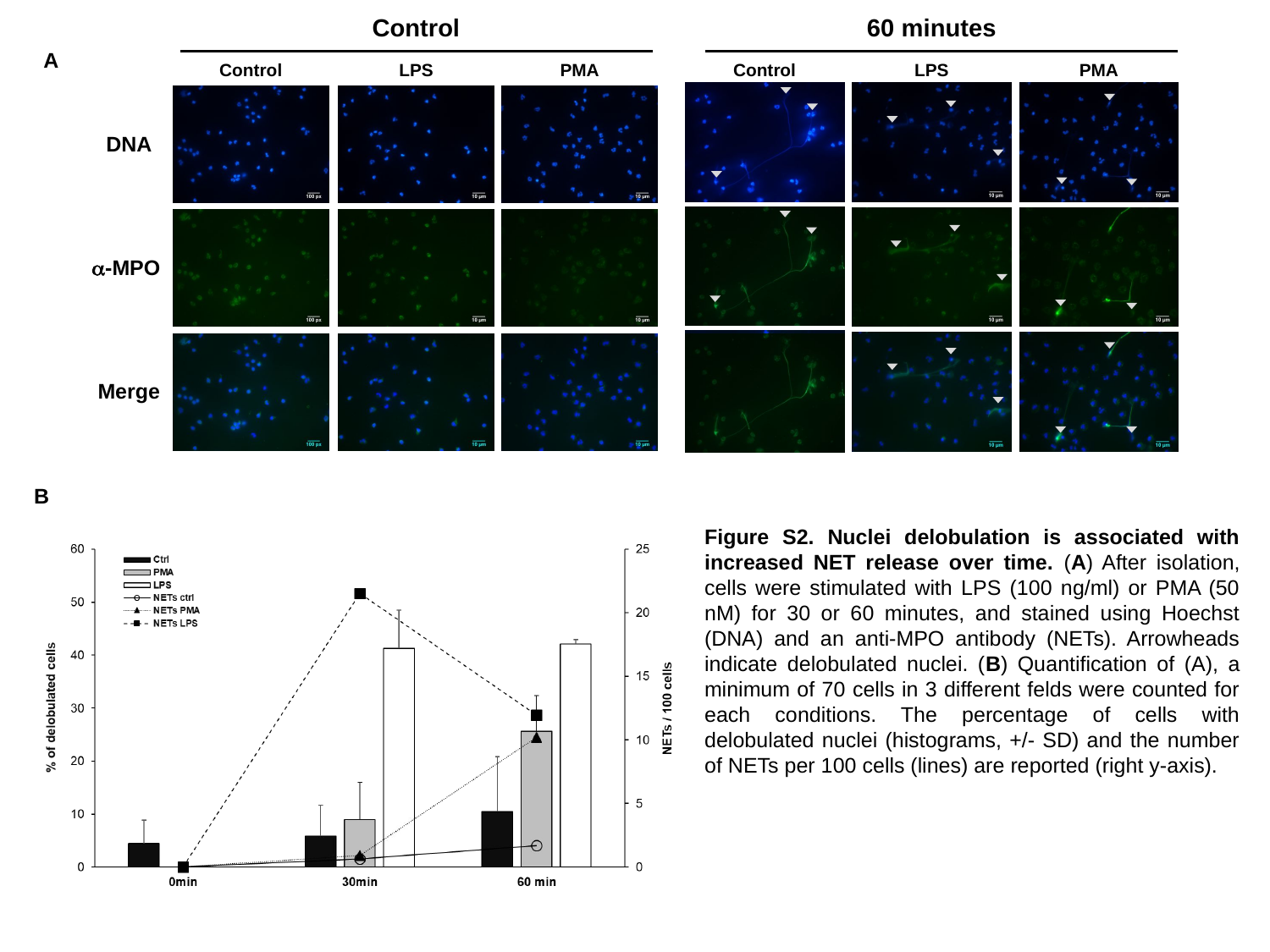

60 minutes
Control
A
Control
LPS
PMA
Control
LPS
PMA
DNA
a-MPO
Merge
B
Figure S2. Nuclei delobulation is associated with increased NET release over time. (A) After isolation, cells were stimulated with LPS (100 ng/ml) or PMA (50 nM) for 30 or 60 minutes, and stained using Hoechst (DNA) and an anti-MPO antibody (NETs). Arrowheads indicate delobulated nuclei. (B) Quantification of (A), a minimum of 70 cells in 3 different felds were counted for each conditions. The percentage of cells with delobulated nuclei (histograms, +/- SD) and the number of NETs per 100 cells (lines) are reported (right y-axis).

## Slide 3
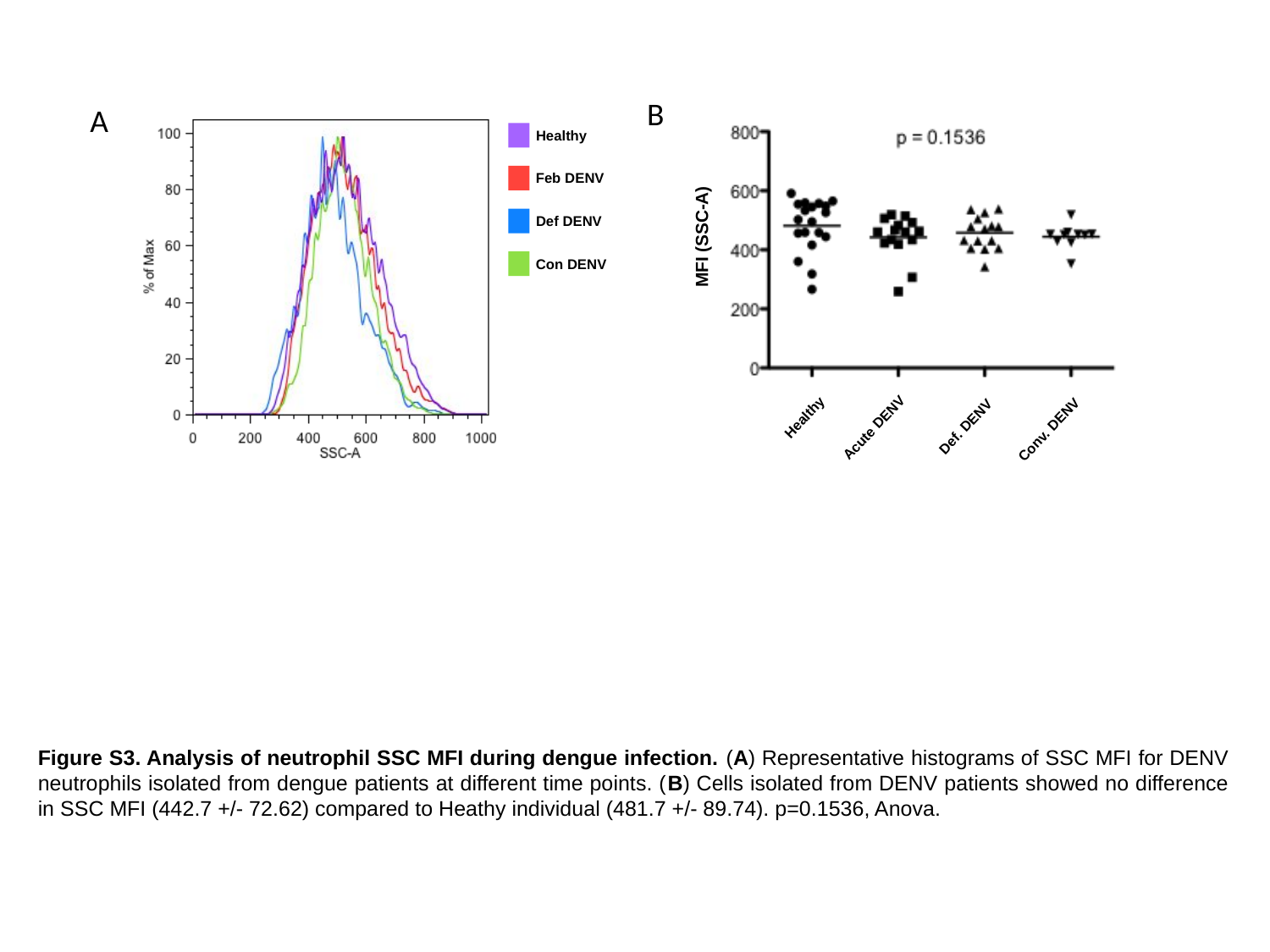

B
A
MFI (SSC-A)
Healthy
Def. DENV
Acute DENV
Conv. DENV
Healthy
Feb DENV
Def DENV
Con DENV
Figure S3. Analysis of neutrophil SSC MFI during dengue infection. (A) Representative histograms of SSC MFI for DENV neutrophils isolated from dengue patients at different time points. (B) Cells isolated from DENV patients showed no difference in SSC MFI (442.7 +/- 72.62) compared to Heathy individual (481.7 +/- 89.74). p=0.1536, Anova.

## Slide 4
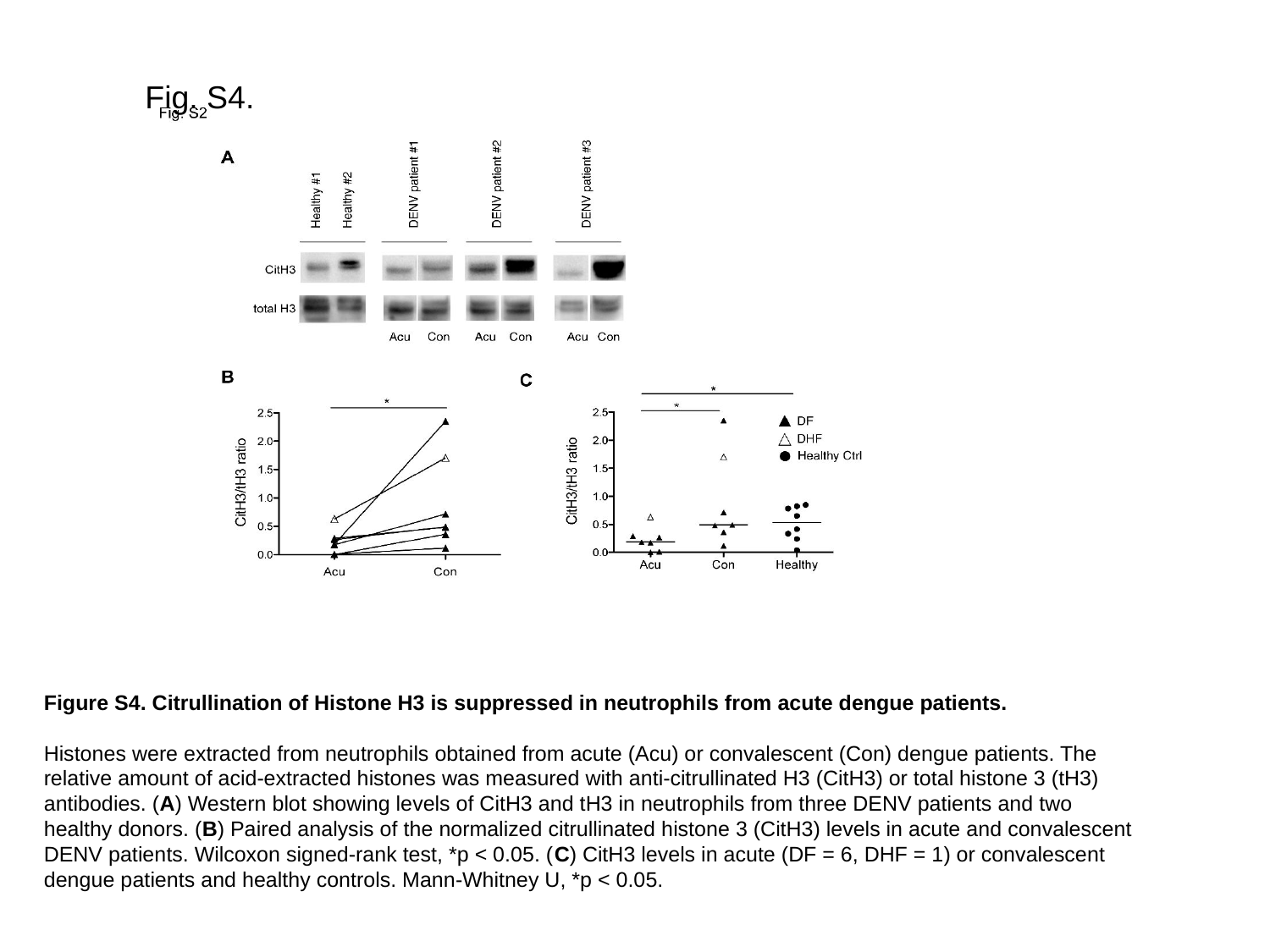

Fig. S4.
Figure S4. Citrullination of Histone H3 is suppressed in neutrophils from acute dengue patients.
Histones were extracted from neutrophils obtained from acute (Acu) or convalescent (Con) dengue patients. The relative amount of acid-extracted histones was measured with anti-citrullinated H3 (CitH3) or total histone 3 (tH3) antibodies. (A) Western blot showing levels of CitH3 and tH3 in neutrophils from three DENV patients and two healthy donors. (B) Paired analysis of the normalized citrullinated histone 3 (CitH3) levels in acute and convalescent DENV patients. Wilcoxon signed-rank test, *p < 0.05. (C) CitH3 levels in acute (DF = 6, DHF = 1) or convalescent dengue patients and healthy controls. Mann-Whitney U, *p < 0.05.

## Slide 5
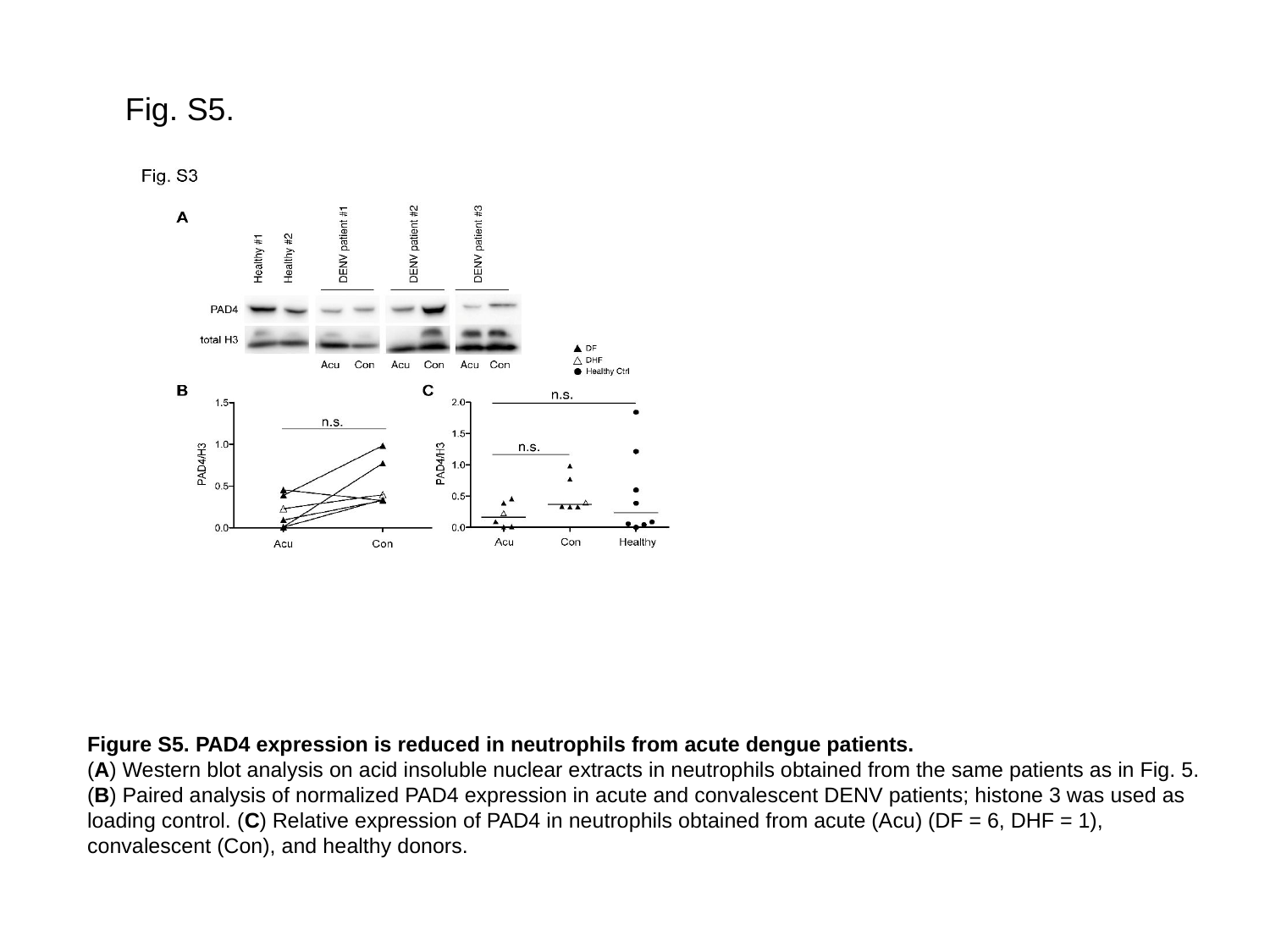

Fig. S5.
Figure S5. PAD4 expression is reduced in neutrophils from acute dengue patients.
(A) Western blot analysis on acid insoluble nuclear extracts in neutrophils obtained from the same patients as in Fig. 5. (B) Paired analysis of normalized PAD4 expression in acute and convalescent DENV patients; histone 3 was used as loading control. (C) Relative expression of PAD4 in neutrophils obtained from acute (Acu) (DF = 6, DHF = 1), convalescent (Con), and healthy donors.

## Slide 6
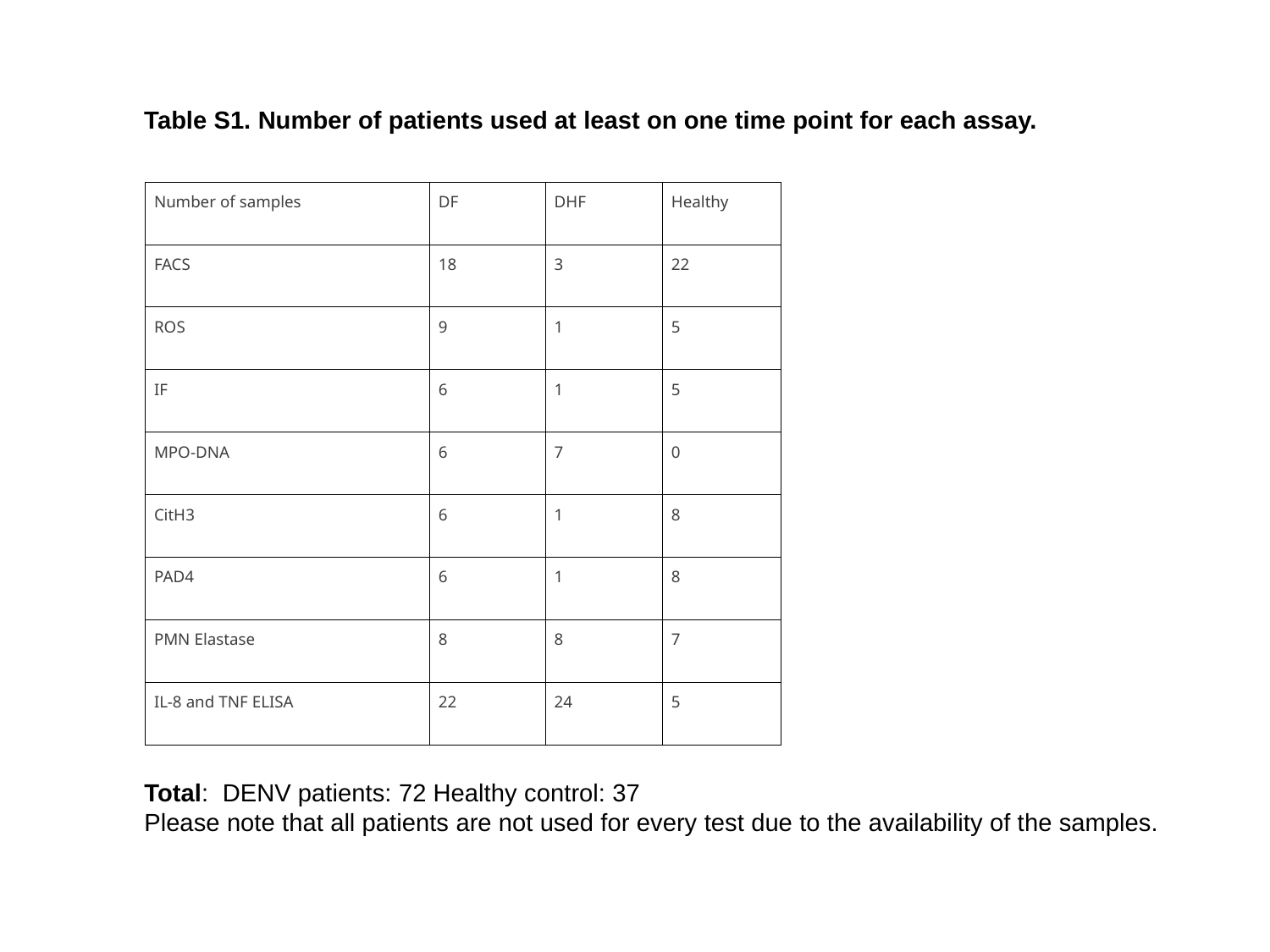

Table S1. Number of patients used at least on one time point for each assay.
| Number of samples | DF | DHF | Healthy |
| --- | --- | --- | --- |
| FACS | 18 | 3 | 22 |
| ROS | 9 | 1 | 5 |
| IF | 6 | 1 | 5 |
| MPO-DNA | 6 | 7 | 0 |
| CitH3 | 6 | 1 | 8 |
| PAD4 | 6 | 1 | 8 |
| PMN Elastase | 8 | 8 | 7 |
| IL-8 and TNF ELISA | 22 | 24 | 5 |
Total:  DENV patients: 72 Healthy control: 37
Please note that all patients are not used for every test due to the availability of the samples.
